# Multiplex PCR for bacterial, viral and protozoal pathogens in persistent diarrhoea or persistent abdominal pain in Côte d’Ivoire, Mali and Nepal

**DOI:** 10.1038/s41598-024-60491-y

**Published:** 2024-05-13

**Authors:** Jasmin K. Jasuja, Florian Bub, Jonas Veit, Hassan K. M. Fofana, Moussa Sacko, Rénion Saye, Justin K. Chatigre, Eliézer K. N’Goran, Joel A. Yao, Basudha Khanal, Kanika Koirala, Narayan R. Bhattarai, Suman Rijal, Lutz von Müller, Emmanuel Bottieau, Marleen Boelaert, François Chappuis, Katja Polman, Jürg Utzinger, Sören L. Becker

**Affiliations:** 1 Institute of Medical Microbiology and Hygiene, Saarland University, Kirrberger Straße, Building 43, 66421 Homburg/Saar, Germany; 2 Department for Infectious Diseases, University Hospital Heidelberg, Heidelberg, Germany; 3 Institut National de Recherche en Santé Publique, Bamako, Mali; 4 Hôpital Mêthodiste de Dabou, Dabou, Côte d’Ivoire; 5 Unité de Formation et de Recherche Biosciences, Université Félix Houphouët-Boigny, Abidjan, Côte d’Ivoire; 6 Centre Suisse de Recherches Scientifiques en Côte d’Ivoire, Abidjan, Côte d’Ivoire; 7 Department of Microbiology, B P Koirala Institute of Health Sciences, Dharan, Nepal; 8 Department of Internal Medicine, B P Koirala Institute of Health Sciences, Dharan, Nepal; 9 Institute for Laboratory Medicine, Microbiology and Hygiene, Christophorus Kliniken, Coesfeld, Germany; 10 Department of Clinical Sciences, Institute of Tropical Medicine, Antwerp, Belgium; 11 Department of Public Health, Institute of Tropical Medicine, Antwerp, Belgium; 12 Division of Tropical and Humanitarian Medicine, Geneva University Hospitals, Geneva, Switzerland; 13 Swiss Tropical and Public Health Institute, Allschwil, Switzerland; 14 University of Basel, Basel, Switzerland

**Keywords:** Infectious-disease diagnostics, Public health, Clinical microbiology, Infectious diseases

## Abstract

In contrast to acute diarrhoea, the aetiology of persistent digestive disorders (≥ 14 days) is poorly understood in low-resource settings and conventional diagnostic approaches lack accuracy. In this multi-country study, we compared multiplex real-time PCR for enteric bacterial, parasitic and viral pathogens in stool samples from symptomatic patients and matched asymptomatic controls in Côte d’Ivoire, Mali and Nepal. Among 1826 stool samples, the prevalence of most pathogens was highest in Mali, being up to threefold higher than in Côte d’Ivoire and up to tenfold higher than in Nepal. In all settings, the most prevalent bacteria were EAEC (13.0–39.9%) and *Campylobacter* spp. (3.9–35.3%). *Giardia* *intestinalis* was the predominant intestinal protozoon (2.9–20.5%), and adenovirus 40/41 was the most frequently observed viral pathogen (6.3–25.1%). Significantly different prevalences between symptomatic and asymptomatic individuals were observed for *Campylobacter*, EIEC and ETEC in the two African sites, and for norovirus in Nepal. Multiple species pathogen infection was common in Côte d’Ivoire and Mali, but rarely found in Nepal. We observed that molecular testing detected multiple enteric pathogens and showed low discriminatory accuracy to distinguish between symptomatic and asymptomatic individuals. Yet, multiplex PCR allowed for direct comparison between different countries and revealed considerable setting-specificity.

## Introduction

Digestive disorders rank among the leading causes of morbidity and mortality worldwide, with diarrhoea playing the most important role^1^. Although mortality has declined considerably over the past decades, diarrhoea still ranks as the fourth leading cause of death in children under the age of 5 years^2^. Globally, an estimated 1.65 million people die from diarrhoea annually, among them 446,000 children before they reach their fifth birthday^2^. The highest burden of digestive disorders is observed in low- and middle-income countries (LMICs), exacerbated by lack of clean water, poor sanitation and hygiene standards^1,3^. Compared to acute diarrhoea (defined as passage of three or more loose or liquid stools per day that lasts several hours to 13 days), less attention has been paid to persistent diarrhoea (defined as any diarrhoea lasting at least 14 days) and persistent abdominal pain (defined as self-reported, aetiologically unclear abdominal pain in children and adolescents aged 1–18 years). The few well-designed studies revealed a complex aetiology with considerable local idiosyncrasies^4–7^. Indeed, it is impossible to identify the causative agent solely based on the clinical symptomatology, and sensitive laboratory examinations are required to determine the causative agents of persistent digestive disorders^6,8^. However, the most widely used conventional methods are insensitive and only target a narrow spectrum of pathogens. Molecular diagnostic methods, such as multiplex real-time PCR, are gaining importance^6^. Multi-centred studies may provide new insights into key pathogens in different geographical settings and might help to select the most useful diagnostic tests^9^.

In order to investigate the aetiology of persistent digestive disorders in LMICs, the Neglected Infectious diseases DIAGnosis (NIDIAG) research network was set up and run for 5 years with funding from the European Commission^10,11^. Within NIDIAG, we designed a case–control study and examined stool samples in two African and one Asian site. One NIDIAG study was designed to prospectively investigate the aetiology of persistent digestive disorders. Here, we report on a *post-hoc* molecular analysis carried out on preserved stool samples stemming from both symptomatic patients and asymptomatic controls. The aims of this investigation were to comparatively describe the aetiological spectrum of persistent digestive disorders in the different settings, and to assess whether a threshold to identify pathogens with clinical significance can be identified.

## Methods

### Ethics statement

The protocol for this NIDIAG multi-country study was approved by the ethics committees of the University of Antwerp in Belgium, the ‘Ethikkommission beider Basel’ (EKBB) in Basel, Switzerland, the ﻿‘Institut National de Recherche en Santé Publique﻿’ in Bamako, Mali, the Ministry of Health in Abidjan in Côte d’Ivoire and the Nepal Health Research Council in Nepal. This study is registered on ClinicalTrials.gov (identifier: NCT02105714) and a detailed study protocol has been published elsewhere^12^.

### Study area, design and population

The original NIDIAG study was designed as a prospective case–control study pertaining to persistent digestive disorders, which are defined as persistent diarrhoea (≥ 14 days) in individuals aged ≥ 1 year and/or persistent abdominal pain (≥ 14 days) in infants and adolescents aged 1–18 years. Persistent diarrhoea was defined according to World Health Organization (WHO) guidelines, i.e. three or more liquid bowel movements per day lasting ≥ 14 days. Persistent abdominal pain was defined as localised or diffuse abdominal pain ≥ 14 days with possible intermittence. Sample size calculations for the clinical study sites were based on available data on the frequency of persistent digestive disorders in published studies from resource-constrained areas^12^.

The study in Mali was carried out in the district reference healthcare centre based in Niono, a sub-regional administrative town, approximately 300 km northeast of Mali’s capital Bamako from August 2014 to May 2015. In Côte d’Ivoire, the study site was a regional reference hospital (Hôpital Méthodiste) in Dabou, a medium-sized city approximately 30 km west of the country’s economic capital Abidjan. Participants were enrolled between October 2014 and December 2015. In Nepal, the study was carried out at the B P Koirala Institute of Health Sciences in Dharan, a town approximately 350 km southeast of the country’s capital Kathmandu, and at the Dhankuta District Hospital in the town of Dhankuta, situated approximately 50 km north of Dharan. Sample collection in Nepal was carried out between July 2014 and August 2015.

Recruitment was conducted as previously described^13^. To each enrolled patient, one control without any gastrointestinal complaints in the 2 months preceding enrolment was matched by age group, sex and geographical location of residence. The reason to include matched asymptomatic controls in a 1:1 ratio to symptomatic patients was to investigate the aetiology of persistent digestive disorders and to provide discriminative data on the distribution of pathogens in order to enhance interpretation of causal associations.

### Field and laboratory procedures

A series of conventional bacteriological and parasitological tests was carried out in the different settings, which have been described in detail elsewhere^13^. For the molecular diagnostic investigation reported here, participants provided stool samples in pre-labelled containers of which 500 mg of solid or 500 µl of fluid sample was transferred into 1 ml Eppendorf tubes. The samples were gently vortexed with 1–2 ml of 96% ethanol and stored in a fridge at 4 °C^14^. In all study countries, the samples were transferred within a few days from the study site to regional diagnostic centres, where they were stored in freezers at − 20 °C. At the end of the study (after a maximum storage period of up to 14 months), all samples were transferred on dry ice to the Institute of Medical Microbiology and Hygiene in Homburg, Germany, pending molecular diagnostic tests adhering to standard operating procedures. All research activities and microbiological diagnostic tests were performed in accordance with relevant guidelines and regulations.

### Molecular post-hoc testing

At the Institute of Medical Microbiology and Hygiene in Homburg, 200 μl of each sample was taken of the 500 μl aliquots for automated nucleic acid extraction with the Promega Maxwell® 16 instrument (Promega Corporation; Fitchburg, United States of America). All samples were purified with the tissue LEV Blood DNA Purification Kit by the same manufacturer, and 1 µl of internal control RNA (for the viral stool panel) or DNA (for the bacterial and viral stool panel) was added. This purification kit was recommended by the manufacturer based on own testing protocols. However, at study inception, we comparatively assessed ten samples using the LEV Blood DNA Purification Kit and the LEV Blood RNA Purification Kit, but did not observe any differences with regard to the subsequent detection of RNA viruses. After extraction, multiplex real-time PCR was carried out on a Stratagene Mx3005P instrument, adhering to the supplier’s instructions (R-Biopharm AG; Darmstadt, Germany).

Regarding bacterial and parasitic infections, the current study employed the following, commercially available multiplex PCR kits from R-Biopharm (https://clinical.r-biopharm.com/): RIDA®GENE Bacterial Stool Panel (for detection of *Salmonella* spp.*, Campylobacter* spp. and *Yersinia enterocolitica*)*,* RIDA®GENE EAEC Stool Panel (for enteroaggregative *Escherichia coli*), RIDA®GENE ETEC/EIEC Stool Panel (for enteroinvasive *E. coli* (EIEC)/*Shigella* spp. (which are both characterised by the presence of *ipaH*) and enterotoxigenic *E. coli* (ETEC) with the subtypes LT and ST) and the RIDA®GENE Parasitic Stool Panel I (targeting *Cryptosporidium* spp., *Dientamoeba fragilis*, *Entamoeba histolytica* and *Giardia intestinalis* (synonymous: *G.* *lamblia*, *G. duodenalis*)). In brief, 5 μl of each extracted sample was added to a PCR mix containing 19.9 μl of reaction mix and 0.1 μl of Taq polymerase.

For detection of viral nucleic acids, the RIDA®GENE Viral Stool Panel I for adenovirus 40/41 (target gene: hexon), astrovirus (target gene: CAP), norovirus (target gene: ORF1/ORF2 junction region) and rotavirus (target gene: NSP3) was employed. Five µl of each sample extraction was added to 20 µl of master mix comprising 12.5 µl of reaction mix, 6.9 µl of primer–probe-mix and 0.7 µl of enzyme mix. Negative and positive controls for each stool panel were enclosed with 1 µl of internal control RNA (ICR) and 1 μl of internal control DNA (ICD), respectively, and were part of each PCR run. Samples were analysed using MxPro® QPCR Data Analysis Software.

### Infection intensity

We determined infection intensities based on median cycle threshold (C_t_) values obtained by real-time PCR analysis per pathogen, that were stratified by cases and controls and classified into four groups: (i) high intensity (C_t_ ≤ 24.9); (ii) medium (C_t_ 25.0–29.9); (iii) low (C_t_ 30.0–34.9); and (iv) very low (C_t_ ≥ 35.0) infection intensity.

### Statistical analysis

Results of multiplex real-time PCR for each study country were tabulated in a Microsoft Excel file, version 14.0 (edition 2010, Microsoft Corporation). Prevalence and co-infections were calculated with Microsoft Excel, while distributional differences were assessed by Pearson’s χ^2^. A p-value of < 0.05 was considered statistically significant. All stool samples of sufficient quantity to perform multiplex PCR were considered for further analysis.

## Results

### Study cohort

A total of 1826 stool samples were collected, including 916 subjects with persistent diarrhoea and/or persistent abdominal pain and 910 matched controls. The study cohorts in Mali, Côte d’Ivoire and Nepal comprised 1100 (553 cases and 547 controls), 519 (260 cases and 259 controls) and 207 (103 cases and 104 controls) individuals, respectively. Details on the predominant symptomatology and the age distribution in the study sites are presented in Table 1.

**Table 1:** Epidemiological baseline information and occurrence of symptomatic cases with persistent diarrhoea (with a duration ≥ 14 days; in individuals aged ≥ 1 year) and/or persistent abdominal pain (with a duration ≥ 14 days; only in individuals aged 1–18 years) and asymptomatic controls in a case–control study to investigate persistent digestive disorders in Côte d’Ivoire, Mali and Nepal.

Study sites	N of analysed samples	Cases				Controls	Sex		Age (years)	
		n	Abdominal pain	Persistent diarrhoea	Both		Female	Male	Median	Range
Côte d’Ivoire	519	260	146	77	37	259	246	273	9	1–75
Mali	1100	553	542	11	0	547	568	532	9	1–56
Nepal	207	103	26	64	13	104	91	116	28	1–86

### Bacterial, parasitic and viral infections

EAEC was the predominant bacterium in all study sites, with an overall prevalence of 39.9% in Mali, 21.6% in Côte d’Ivoire and 13.0% in Nepal, followed by *Campylobacter* spp. (Table 2). *Campylobacter* spp.*,* EIEC/*Shigella* spp. and ETEC were significantly more frequent in patients than in controls at the two African sites, while this was observed for EAEC only in Côte d’Ivoire. No statistically significant difference between cases and controls was observed for any bacteria in Nepal. *Salmonella* and *Yersinia* were only very rarely found in the entire study cohort (≤ 1%).

**Table 2:** Overall prevalence of bacteria, parasites and viruses in a case–control study conducted in Mali (n = 1100; 553 cases and 547 controls), Côte d’Ivoire (n = 519; 260 cases and 259 controls) and Nepal (n = 207; 103 cases and 104 controls) to investigate the aetiology of persistent digestive disorders (≥ 14 days).

Pathogen	Mali							Côte d’Ivoire							Nepal						
	Total		Cases		Controls		p	Total		Cases		Controls		p	Total		Cases		Controls		p
	N	%	n	%	n	%		n	%	n	%	n	%		n	%	n	%	n	%	
All samples	1100		553		547			519		260		259			207		103		104		
Bacteria																					
EAEC	439	39.9	224	40.5	215	39.3	0.165	112	21.6	67	25.9	45	17.3	**0.013**	27	13.0	14	13.6	13	12.5	0.816
*Campylobacter* spp.	388	35.3	214	38.7	174	31.8	**0.017**	92	17.7	57	22.0	35	13.5	**0.011**	8	3.9	3	2.9	5	4.8	0.479
EIEC/*Shigella* spp.	170	15.5	100	18.1	70	12.8	** < 0.001**	53	10.2	35	13.5	18	6.9	**0.013**	5	2.4	4	3.9	1	1.0	0.171
ETEC LT	130	11.8	82	14.8	48	8.8	**0.002**	48	9.2	32	2.6	16	6.2	**0.015**	2	1.0	1	1.0	1	1.0	0.995
ETEC ST	37	3.4	22	4.0	15	2.7	0.256	11	2.1	8	3.1	3	1.2	0.126	0	0	0	0.0	0	0.0	–
*Salmonella* spp.	8	0.7	2	0.4	6	1.1	0.151	1	0.2	1	0.4	0	0.0	0.316	2	1.0	1	1.0	1	1.0	0.995
*Yersinia* spp.	1	≤ 0.1	1	0.2	0	0.0	0.990	0	0.0	0	0.0	0	0.0	–	0	0	0	0.0	0	0.0	–
Parasites																					
*Giardia intestinalis*	225	20.5	151	27.3	74	13.5	** < 0.001**	60	11.6	36	13.9	24	9.2	0.096	6	2.9	3	2.9	3	2.9	0.990
*Dientamoeba fragilis*	179	16.3	74	13.4	105	19.2	**0.015**	37	7.1	22	4.2	15	5.8	0.228	14	6.8	4	3.9	10	9.6	0.101
*Cryptosporidium* spp.	46	4.2	20	3.6	26	4.8	0.346	11	2.1	8	3.1	3	1.2	0.126	0	0	0	0.0	0	0.0	–
*Entamoeba histolytica*	9	0.8	6	1.1	3	0.5	0.323	5	1.0	1	0.4	4	1.5	0.179	0	0	0	0.0	0	0.0	–
Viruses^a^																					
Adenovirus 40/41	240	25.1	101	23.3	139	26.5	0.256	91	17.6	60	23.3	31	12.0	**0.001**	13	6.3	8	7.8	5	4.8	0.380
Norovirus	91	9.5	33	7.6	58	11.1	0.704	40	7.8	22	8.6	18	6.9	0.486	11	5.3	9	8.7	2	1.9	**0.029**
Astrovirus	31	3.2	9	2.1	22	4.2	0.065	11	2.1	10	3.9	1	0.4	**0.006**	0	0	0	0.0	0	0.0	–
Rotavirus	16	1.7	10	2.3	6	1.1	0.162	8	1.6	7	2.7	1	0.4	**0.031**	3	1.4	3	2.9	0	0.0	–

^a^Due to PCR inhibition events, the n of analysed samples for viral pathogens was slightly lower than for bacterial and parasitic agents, i.e., in Mali 433 cases and 524 controls, in Côte d’Ivoire 257 cases and 259 controls, and in Nepal 103 cases and 104 controls.

Significant values are in bold.

Among the intestinal protozoa, *G. intestinalis* and *D. fragilis* were frequently detected in Mali and Côte d’Ivoire, with *G. intestinalis* being significantly more prevalent in symptomatic patients. In all study sites, the prevalence of *D. fragilis* was higher among controls, though a statistical significance was only observed among participants in Mali. In Nepal, the overall prevalence of *G. intestinalis* and *D. fragilis* was below 7% and no infections with *Cryptosporidium* spp. or *E. histolytica* were observed.

Adenovirus was the most frequent viral infection, with particularly high prevalence in the two African sites. In Côte d’Ivoire, the prevalence of the different viruses was significantly elevated in cases as compared to controls, except for norovirus. In Nepal, only norovirus was significantly more prevalent in symptomatic patients. Rotavirus and astrovirus were rare in all three settings (≤ 5%).

**Figure 1:**
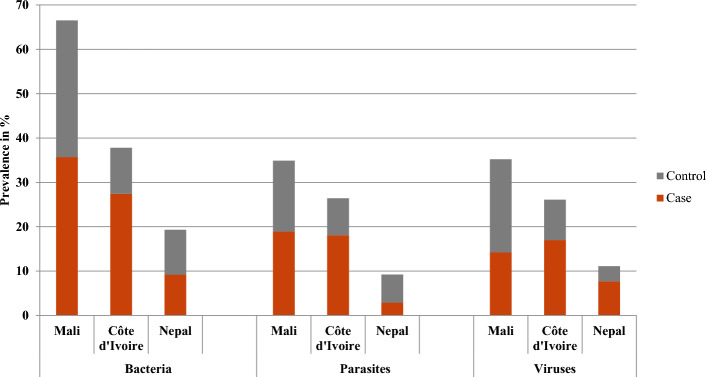
Overall prevalence of patients with bacterial, parasitic and viral infections in a multi-centre case–control study on persistent digestive disorders, as defined as persistent diarrhoea (≥ 14 days; individuals aged 1 year and older) and persistent abdominal pain (≥ 14 days; children and adolescents aged 1–18 years) conducted in Mali, Côte d’Ivoire and Nepal.

The prevalence of most pathogens was highest in Mali, being 1.5- to 3-fold higher than in Côte d’Ivoire and 3- to 10-fold higher than in Nepal. Considering any bacterial infection, the prevalence in Mali was 67.1% as compared to 45.5% in Côte d’Ivoire and 19.3% in Nepal, with similar country-specific patterns regarding the overall prevalence of parasitic and viral infection (Fig. 1).

### Infection intensity

The majority of infections was of rather low infection intensity, as identified by C_*t*_ values. Except for EIEC/*Shigella* spp., *Salmonella*, norovirus, rotavirus and astrovirus*,* infection intensity of most pathogens was not significantly different between cases and controls in the different study sites. Details are presented in a heat map in Table 3.

**Table 3:**
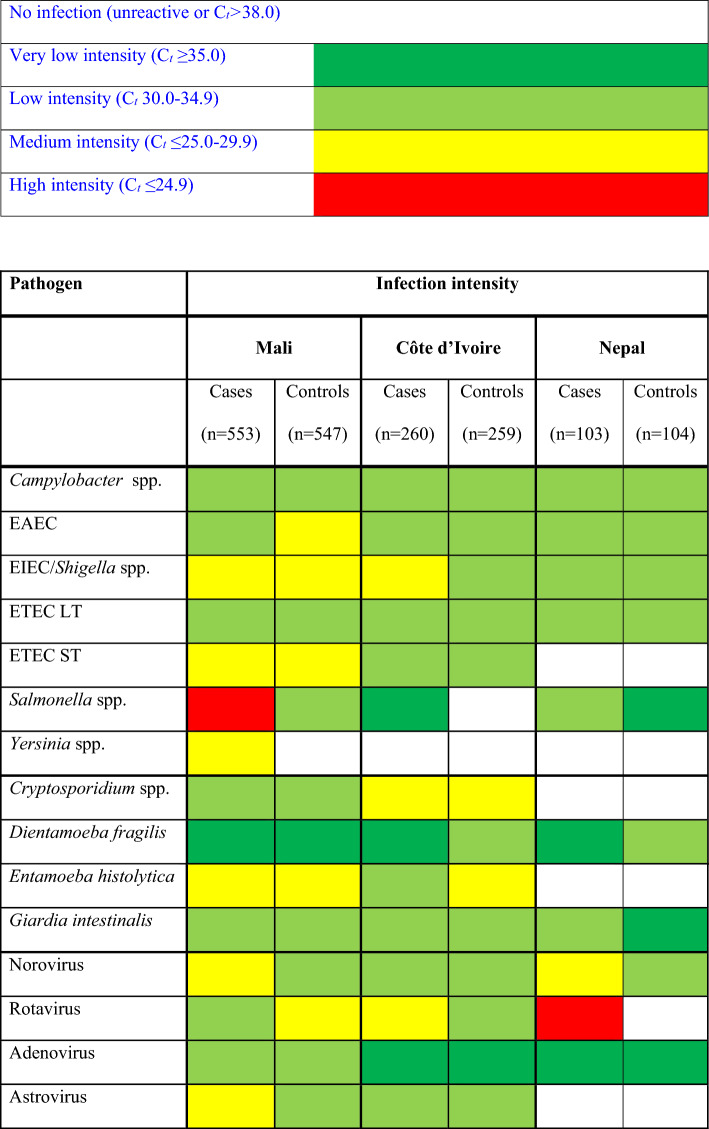
Heat map of infection intensities as obtained by real-time PCR median cycle threshold (C_*t*_) values in a study on enteric pathogens in patients with persistent digestive disorders (≥ 14 days) and matched controls from Côte d’Ivoire, Mali and Nepal.

### Co-infections

**Figure 2:**
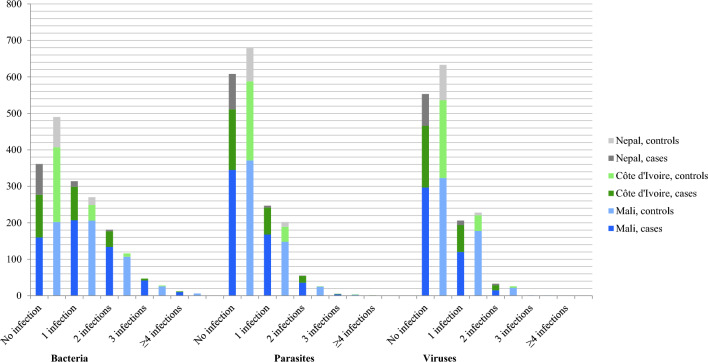
Concurrent infections with bacterial, parasitic and viral pathogens in symptomatic cases and asymptomatic controls in a multi-centre study on persistent digestive disorders (≥ 14 days) in Mali (n = 1100), Côte d’Ivoire (n = 519) and Nepal (n = 207).

Co-infections were rare in Nepal, but common in the two African sites, particularly pertaining to bacterial co-infections among cases (Fig. 2). No clear trend towards distinct differences between the number of overall co-infections and the presence or absence of symptoms was found.

## Discussion

In this study, we found that the prevalence of most pathogens investigated, including co-infection rate, was highest in Mali, followed by Côte d’Ivoire. The study setting in Nepal showed the lowest prevalence for individual pathogens and concurrent infections among the three sites. In all settings, the most prevalent bacteria were EAEC and *Campylobacter* spp., while *G.* *intestinalis* was the predominant parasite and adenovirus the predominant viral pathogen. In the two African sites, *Campylobacter*, EIEC/*Shigella* spp. and ETEC were significantly more prevalent in symptomatic patients than asymptomatic controls. Co-infections with two or more pathogens were common in the two African sites, but absent in Nepal. Most infection intensities were low.

Even though remarkable progress has been made to reduce diarrhoeal diseases and associated morbidity, diarrhoea remains a leading cause of death in all age groups, with children under the age of 5 years most severely affected^2,15^, in particular due to resulting growth faltering, undernutrition and immunosuppression^16^. Additionally, the burden of persistent abdominal pain has not yet been thoroughly addressed in large clinical studies. Indeed, these long-term effects might account for more morbidity than the actual acute health effects, as previously shown for cryptosporidiosis^17^. Sanitation constitutes one of the major modifiable risk factors for diarrhoeal diseases^18^ and improved access to water, sanitation and hygiene (WASH) correlates with mortality reductions in most studies^19^. The link between poverty and infectious diseases has been specifically addressed for parasitic diseases^20^. At the country level, researchers observed a correlation between the human development index (HDI; a measure that is calculated based on the average healthy life span, level of education and standard of living) and the ‘worm index’ (i.e. the number of individuals requiring preventive chemotherapy with anthelminthic drugs against intestinal helminth infections, schistosomiasis and lymphatic filariasis divided by the total population in this country)^21^. A strong inverse correlation between HDI and worm index was observed, which might illustrate the long-term negative impact of these infections on the general well-being, productivity and economic development. According to the most recent HDI, Nepal, Côte d’Ivoire and Mali rank at positions 143, 159 and 186, respectively, among 191 countries listed^22^. Our study corroborates such findings. Indeed, we observed considerably higher prevalences of overall infections, individual pathogens and number of co-infections in countries with a lower HDI (e.g. the prevalence of EAEC was 39.9% in Mali, 21.6% in Côte d’Ivoire and 13.0% in Nepal). Additional research is required to support or refute these associations, and multi-country investigations using the same methodologies across sites are particularly relevant.

Even though the implementation of molecular diagnostics in resource-constrained settings remains challenging, PCR assays are key to identify and attribute diarrhoeal diseases to their microbiological causes, and have important ramifications for the development of clinical diagnosis-treatment algorithms^23^. From an epidemiological perspective, centralised *post-hoc* analyses of preserved stool samples with highly standardised molecular tests allow for comparable assessments across different sites, regardless of the respective diagnostic capacities in the field. In contrast, our findings underscore that PCR-based approaches alone without an individual clinical assessment cannot accurately differentiate symptomatic patients from asymptomatic controls.

Large research consortia have recently improved our understanding of the aetiology of acute diarrhoeal infections. In the Enteric Infections and Malnutrition and the Consequences for Child Health study (MAL-ED) in rural areas of South America, Africa and Asia, similar results pertaining to the prevalence of *Campylobacter* spp., EAEC and *G. intestinalis* in symptomatic and asymptomatic infants were found^24^. In the Global Enteric Multicenter Study (GEMS), a case–control study conducted in sub-Saharan Africa and South Asia, *Campylobacter* spp. was identified as a major cause of moderate-to-severe diarrhoea^5,9^. In contrast to our study, in which *Cryptosporidium* spp. was not highly prevalent, cryptosporidiosis was identified as one of the most significant infections in the MAL-ED, GEMS and VIDA studies^24–27^. However, a direct comparison between our study and the GEMS and MAL-ED studies is difficult, as the majority (77.9%) of all patients presenting with persistent digestive disorders in our investigation were children and adolescents presenting with persistent abdominal pain rather than diarrhoea as the main symptomatology. Indeed, the actual etiologies of persistent abdominal pain and diarrhoea are poorly recognised and likely to be multifactorial^28^. Yet, persistent diarrhoea has recently been acknowledged as a growing public health concern, particularly in sub-Saharan Africa, as evidenced by the VIDA study^29^. The high prevalence of *D. fragilis*, which was significantly more common among asymptomatic controls, further questions its potential pathogenic relevance^30^, although this protozoon was also recently associated with persistent abdominal pain in returning travelers^31^. Recent studies suggested that the severity of symptoms depends on the cumulative number of co-infections rather than on a single causative pathogen. Indeed, several studies did not find convincing evidence of a specific pathogen being associated with persistent diarrhoea in children aged below 6 years in LMICs^32,33^. It might be hypothesised that a primary infection facilitates a subsequent secondary infection with a possibly lower virulent pathogen, and hence, may increase the risk to develop diarrhoea^24,32,34^. The high rate of pathogen detection in asymptomatic children might be explained with residual nucleic acid shedding that may persist for months after previous infections^24^. Quantification of the pathogen load may help to distinguish clinically relevant infections, even though we were unable to define a C_*t*_-based threshold. Longitudinal molecular analyses and parallel bacterial cultures might help to define such clinically useful cut-off values. However, it is important to note that even subclinical PCR findings of pathogens in the stool were recently shown to be associated with considerable long-term negative effects regarding childhood growth and stunting^35^.

In contrast to acute diarrhoea, few research initiatives have targeted persistent diarrhoea and persistent digestive disorders, which were in the focus of the NIDIAG consortium. Indeed, most data stem from single-centre studies in returning travelers^28^. In a recent case–control study from Ethiopia, 14% of children with diarrhoea complained about prolonged or persistent diarrhea, and hence, the authors urged for the rapid development of specific guidelines for these conditions^36^.

Our study has several limitations. First, the molecular analyses were carried out several months after stool collection, and, even though cold chain conditions were meticulously maintained, nucleic acid degradation may have occurred and might have led to lower positivity rates, at least for some of the pathogens investigated. Second, we did not perform bacterial and viral culture to correlate these findings to PCR results. Third, we relied on patients’ self-reports of persistent diarrhoea and persistent abdominal pain. Hence, we cannot ascertain whether the symptomatology had indeed been present for at least 2 weeks in all patients. Fourth, the design of our study does not allow inferring distinct causalities between a specific pathogen and the related clinical symptomology. Fifth, even though our PCR approach covered a wide range of pathogens, other intestinal pathogens (e.g. helminths) are missing. More complex approaches, such as metagenomics or more recently commercialised and more comprehensive multiplex PCR panels, hold promise to provide a more thorough characterisation of the intestinal microbial communities in patients and controls^37^. Sixth, no comprehensive information on previous clinical and preventive measures in participants such as e.g. previous intake of antimicrobials and rotavirus vaccination in early childhood was readily available.

## Conclusions

We provide insights into the role of bacterial, protozoal and viral pathogens, some of which are frequently missed by conventional methods, based on detailed molecular examination of stool samples obtained from patients with persistent digestive disorders and asymptomatic controls in Côte d’Ivoire, Mali and Nepal. We observed high setting-specificity, but *Campylobacter* spp., *G.* *intestinalis* and norovirus were significantly more prevalent among symptomatic patients in most study areas. Our data indicate a correlation between the average pathogen load in the individual sites and the corresponding country HDI, which might be governed by poor WASH. Further studies are warranted to assess the long-term effects of intestinal infections beyond diarrhoeal disease manifestations.

## Data Availability

The datasets are available from the corresponding author on reasonable request.
